# The Influence of Annealing at 500 and 900 °C on the Structure and Mechanical Properties of Al_x_CoCrFeNi Alloys

**DOI:** 10.3390/ma16031245

**Published:** 2023-02-01

**Authors:** Marzena Tokarewicz, Małgorzata Grądzka-Dahlke, Katarzyna Rećko, Magdalena Łępicka

**Affiliations:** 1 Department of Materials and Production Engineering, Faculty of Mechanical Engineering, Bialystok University of Technology, ul. Wiejska 45 C, 15-351 Białystok, Poland; 2 Faculty of Physics, University of Bialystok, K. Ciołkowskiego 1L, 15-245 Białystok, Poland

**Keywords:** high-entropy alloys, Al_x_CoCrFeNi, AlCoCrFeNi, mechanical properties, crystal structure

## Abstract

The AlCoCrFeNi high-entropy alloy is sensitive to heat treatment. The aim of the present study was to test a similar correlation for Al_x_CoCrFeNi alloys with less than equimolar aluminum content. This paper presents a study of the annealing effect on the structure and mechanical properties of selected alloys. Al_x_CoCrFeNi alloys (x = 0, 0.5, 0.7) were fabricated by the induction melting method. The obtained specimens were annealed at 500 °C and 900 °C. A detailed study of the changes in crystalline structure due to annealing was conducted. Three-point bending and hardness tests were carried out for the as-cast and annealed specimens to determine selected mechanical properties. The study confirmed that increasing the aluminum content in the Al_x_CoCrFeNi alloy improves mechanical properties. For the alloy with aluminum content x = 0.7, hardness increased by 187% and yield strength by 252% compared to the alloy without aluminum. A significant effect of annealing on the crystalline structure of the Al_0.7_CoCrFeNi alloy was found, but this was not followed by changes in mechanical properties.

## 1. Introduction

Traditional alloys consist of one or two main components and contain alloying additives to improve their properties [1]. High-entropy alloys (HEAs) were first described in 2004 [2], so it is a relatively new generation of materials. They contain 5 to 13 elements in amounts ranging from 5 to 35 at.% [3]. HEAs exhibit much better properties than conventional alloys, which will help to overcome many barriers and challenges that are generated by the rapid development of technology. This new group of materials may have high corrosion resistance, yield strength, or hardness, as well as many other useful properties in construction [4,5]. Due to their unique properties, high-entropy alloys can be used in many areas such as the energy and transportation industries, which require many low-density and high-strength components [6]. Since HEAs were developed, many high-entropy alloy systems have been characterized in the literature, e.g., TiNbTaZrMo [7], FeNiMnCr [8], CuCoNiCrAlFeTiV [9], and AlCoCrCuFeNiSi [10].

AlCoCrFeNi was one of the first studied high-entropy alloys and is now one of the most popular. Researchers often study the mechanical properties of the AlCoCrFeNi alloy and the influence of the varying amounts of elements on its properties [11,12,13]. Among all alloy components, aluminum shows the largest influence on the structure and properties. According to the research, depending on the aluminum content, the phases occurring in the alloy change from fcc into an fcc and bcc mixture to bcc [14,15]. The concentration of aluminum in Al_x_CoCrFeNi also significantly affects its mechanical properties, e.g., increasing the aluminum content in the Al_x_CoCrFeNi alloy improves its strength while decreasing its ductility [16] and positively affects its wear properties [17]. In addition, studies show that the microstructure of Al_x_CoCrFeNi alloy changes with the aluminum content [18].

The properties of high-entropy alloys are also determined by the preparation method, selected manufacturing parameters, and heat treatment. HEAs are most commonly obtained by casting and powder metallurgy. Currently, additive methods are also used. The manufacturing method has an impact on the microstructure and the number of defects present in the resulting sample [19]. The effect of annealing on the high-entropy AlCoCrFeNi alloy was extensively investigated. Liang et al. [20] noticed that changes in the crystal structure of AlCoCrFeNi alloy are present after annealing at temperatures higher than 600 °C. Above 600 °C, precipitation of the fcc phase begins. At 800 °C, a new sigma phase of Fe_0.5_Cr_0.5_ begins to form. The results of Cheng et al. [21] also confirm the changes occurring in the AlCoCrFeNi alloy after annealing. The authors of [22] carried out a detailed study of the equimolar AlCoCrFeNi alloy after annealing at different temperatures. They clearly showed that a sigma phase appears in the 900 °C range, which was not identified below 700 °C or above 1000 °C.

The aim of the present study was to test similar relationships of the effect of characteristic temperatures on changes in structure and mechanical properties for Al_x_CoCrFeNi alloys with aluminum contents lower than equimolar. Al_x_CoCrFeNi (where: x = 0; 0.5; 0.7) alloys were obtained by induction melting in an argon atmosphere. The properties of the obtained samples were investigated in the as-cast state and after annealing at 500 °C and 900 °C. A three-point bending test was performed to determine the mechanical properties. The hardness, microstructure, and the occurring phases in the alloys were investigated.

## 2. Materials and Methods

High-entropy alloys Al_x_CoCrFeNi (x = 0, 0.5, 0.7) were obtained by induction melting in a protective argon atmosphere. Pure metals were melted in a crucible placed in an induction coil. The purity of the raw materials was above 99.9%. The current induced in the charge caused the metallic components to melt and mix. The samples were remelted three times to achieve chemical homogeneity. The liquid alloy was then poured into a copper mold with a diameter of Ø = 32 mm and a height of h = 10 mm and then cooled to ambient temperature. The samples were cut on a wire cutter into the shape of beams with dimensions 24 × 3.5 × 1.5 mm for strength tests and cylinders with a diameter of Ø = 12 mm and a height of h = 5 mm for structural analyses. Next, part of them was annealed at 500 °C or at 900 °C in a tube furnace under a protective argon atmosphere (details in Table 1).

Crystal structures of the samples were analyzed by means of X-ray diffraction (XRD) using an Empyrean Panalytical powder diffractometer (Malvern Panalytical, Malvern, UK), with 40 kV and 30 mA and Cu K*α* radiation, *λ* = 1.540598 Å in the Bragg–Brentano geometry. A 2*θ* range from 20° to 135° was covered using a step of 0.026261° and a count time of 400 s per point. The standard used during X-ray refinements was LaB_6_ C660 crystallizing in a cubic system Pm-3m (space group no. 221). The phase analysis was carried out based on the Inorganic Crystal Structure Database (ICSD) using the HighScore program [23]. During the analysis of the annealing effect on the microstructure for simplicity, we concentrated on the strongest reflections, i.e., (111) FCC and (110) BCC structures. The crystallites (grain) size (D) and microstrain effect (ε) were estimated according to the linear Williamson–Hall method (Table 2).

**Figure 1:**
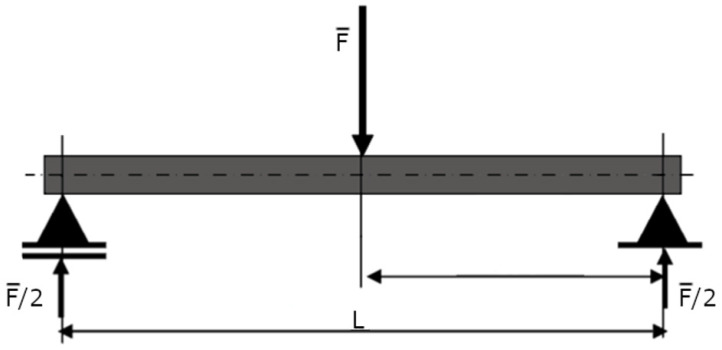
Scheme of the three-point bend test.

For microstructure observation, the samples were ground and polished to a mirror finish, then they were chemically etched in aqua regia. The chemical composition and microstructures were analyzed by a high-resolution scanning electron microscope (SEM-FIB DualBeam Scios 2, Thermo Scientific, Waltham, MA, USA) equipped with an energy-dispersive spectrometer (EDS). Hardness was measured by the Vickers method under a load of 98 N using an INNOVATEST hardness tester (Innovatest Europe BV, Maastricht, The Netherlands). Three-point bending tests were performed on an MTS Insight testing machine (MTS Insight, MTS System Corporation, Eden Prairie, MN, USA). The span length during the examination was 15 mm. To avoid shear load and cutting, 5 mm diameter rollers were used on the press head and for support. A scheme of the three-point bending is illustrated in Figure 1.

The specimens were bent at room temperature with a constant speed of 0.005 mm/s. A load–displacement curve was determined from the obtained data. To determine the conventional bending yield strength *R*_*B*0.2_, it was assumed that the extreme fibers of the specimen elongate by *ε* = 0.2%. For this assumption, the deflection arrow *f_B_*_0.2_, which correlates to such elongation, was calculated using the formula:

(1) $$f_{B0.2}=\frac{\varepsilon L^{2}}{6h},$$

where *ε* = 0.2%, *L* is the span length during the measurement, and *h* is the height of the sample. For the calculated deflection arrow, the load value *P* was read off from the obtained load–displacement diagrams. The value of *R_B_*_0.2_ was then calculated using the formula:

(2) $$R_{B0.2}=\frac{PL}{4W_{g}},$$

where *P* is the load value read from the load–displacement diagram for the calculated value of the deflection arrow *f_B_*_0.2_; *W_g_* is the moment of inertia of the section area about the neutral axis calculated from:

(3) $$W_{g}=\frac{bh^{2}}{6},$$

where *b* is the width of the sample.

To calculate the flexural modulus, the following formula was used:

(4) $$E_{flex}=\frac{F*L^{3}}{d*48*I},$$

where *F* is the applied load, *d* is displacement, and *I* is the moment of inertia calculated from:

(5) $$I=\frac{b*h^{3}}{12}.$$

## 3. Results and Discussion

### 3.1. Homogeneity and Crystal Ordering (Phase Analysis)

Room temperature XRD measurements disclosed several different types of cubic crystal structures. Moreover, a few of them only exhibit a well-ordered state (see Table 2). For better readability, disordered phases are marked with a lower d index. In the case of a complex composition of the sample, the predominant phase is indexed as the “1”, while the residual ones are indexed according to decreasing volume percentage contributions. According to X-ray diagrams, the series of as-cast HEA alloys and those annealed in different temperatures reveal the coexistence of fcc and bcc phases.

**Figure 2:**
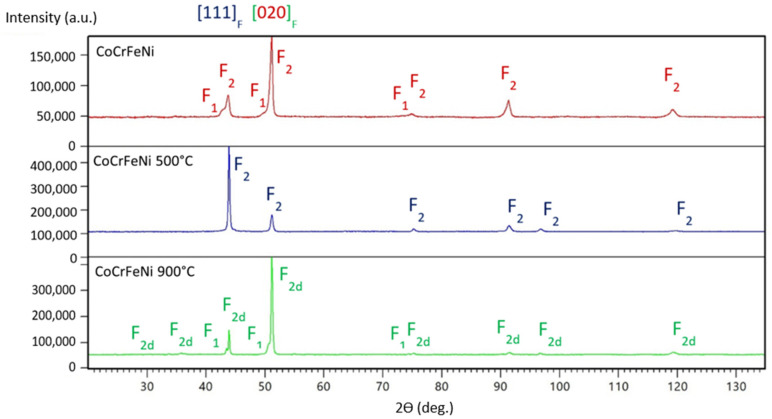
X-ray diagrams of as-cast CoCrFeNi samples and those annealed at different temperatures. The identified phases have been marked according to the graphic symbols described in Table 2. The directions of the mechanical textures are shown at the top of the diagram set.

Figure 2 shows the bi-phase nature of the as-cast CoCrFeNi (red diagram). It is noteworthy that both phases disclose the ordered crystal structure of the face-centered cubic (F) type. The annealing procedure carried out at 500 °C leads to a single-phase product (blue diagram in Figure 2), while the higher annealing temperature results again in a double phase in the sample and a disordered predominant F_2_ structure (green diagram). Neither effect is desired. Moreover, mechanical textures of the [020] type in the double-phased samples and [111] as typical for single-phase sample were discovered.

In both figures below, there are no homogeneous systems in the samples with different aluminum admixtures, either in non-annealed dies or annealed systems. Systems in which face-centered cubic F and body-centered cubic (I) phases coexist are systematically observed.

**Figure 3:**
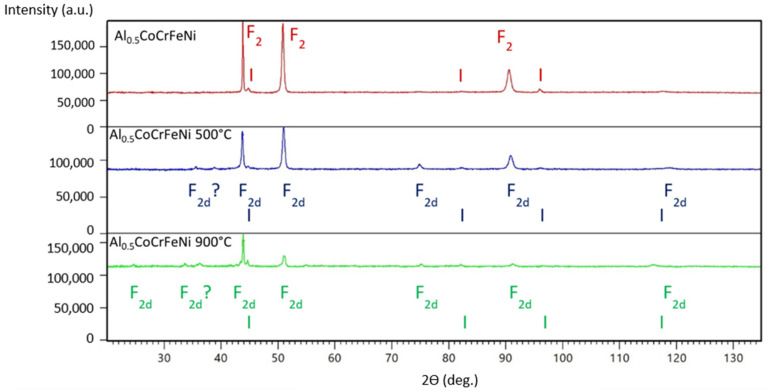
X-ray diagrams of as-cast Al_0.5_CoCrFeNi samples and those annealed at different temperatures. The identified phases have been marked according to the graphic symbols described in Table 2.

The set of X-ray diagrams shown in Figure 3 confirms that all samples are at least double-phase. Moreover, in this series, the mechanical texture is not a problem. It is noteworthy that the Al_0.5_CoCrFeNi sample annealed at 500 °C revealed only traces of a body-centered cubic structure (I).

**Figure 4:**
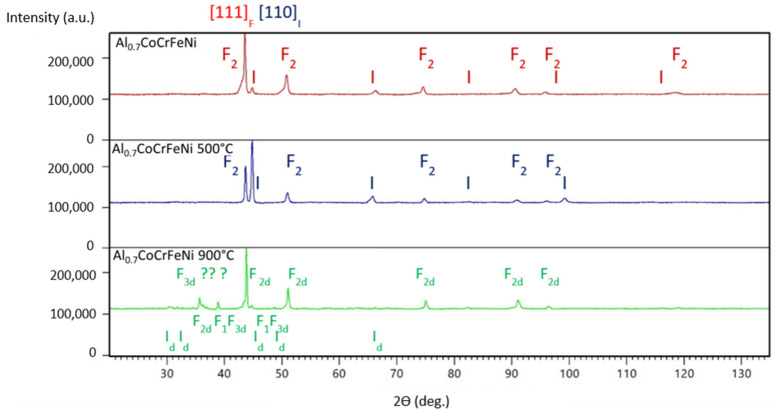
X-ray diagrams of as-cast Al_0.7_CoCrFeNi samples and those annealed at different temperatures. The identified phases have been marked according to the graphic symbols described in Table 2. The directions of the mechanical textures are shown at the top of the diagram set.

Regardless of the history of heat treatment, all samples of the Al_0.7_CoCrFeNi series exhibit the coexistence of fcc and bcc phases (see Figure 4). Undoubtedly, the increase in the annealing temperature of the system leads to an increasingly complex phase distribution. The green diagram in Figure 4 illustrates phase analysis results obtained for Al_0.7_CoCrFeNi annealed at 900 °C. The sample is a multiphase system for which four phases were identified. Several extra peaks remained unrecognized in the range of lowest scattering angles, up to 40 degrees, which can be defined as peaks from the σ-phase, as is often reported for the equimolar alloy AlCoCrFeNi [22]. Moreover, with a limited number of detected intensities, the L2_1_ ordered phase (marked in Table 2 as L) and I_d_, i.e., the disordered body-centered cubic one, become indistinguishable.

In summary, XRD studies show that annealing at 500 °C and 900 °C did not significantly affect the crystalline structure of the x = 0 alloy. These results correspond well with the data in [24]. For the alloy with low aluminum content, Al_0.2_CoCrFeNi, they identified only the presence of the fcc phase, also after annealing at 800 °C and 1000 °C. However, in the case of higher aluminum content, especially for the Al_0.7_CoCrFeNi alloy, additional heat treatment caused significant structural changes. Annealing at 500 °C had a stabilizing effect on the bcc phase compared to the as-cast sample. The percentages of the fcc and bcc phases changed. The data in the literature confirm that an increase in aluminum content causes an increase in the proportion of the bcc phase [25]. However, annealing at 900 °C resulted in a permanent transformation to the fcc structure, which corresponds well with the data obtained by the authors of [26]. The appearance of additional peaks for alloys at aluminum contents x = 0.5 and x = 0.7, which can be defined as the σ-phase, should be noted. Many authors investigating Al_x_CoCrFeNi alloys with higher aluminum content highlight the presence of this phase at temperatures around 800–900 °C, which may affect the mechanical properties of the materials [22,27,28]. However, a detailed analysis of the obtained diffractograms does not indicate the clear presence of the σ-phase. The explanation may be that the aluminum content in the investigated alloys is too low to initiate the transformation with the formation of this phase, which is also confirmed by the results presented in the work [27] for alloys with aluminum contents x = 0.75 and x = 1.25. Similar results were obtained by the authors of [28,29]. It should be pointed out that the authors of [30] also did not observe the σ-phase during annealing of Al_x_CoCrFeNi alloys (x = 0; 0.1; 0.3; 0.5).

### 3.2. Microstructure and Chemical Composition

**Figure 5:**
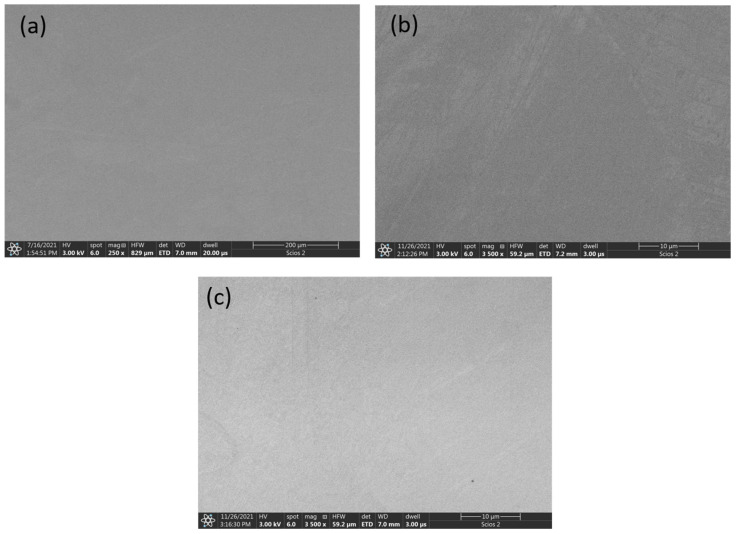
SEM micrographs of CoCrFeNi alloy: (**a**) as-cast, (**b**) annealed at 500 °C, and (**c**) annealed at 900 °C.

Figure 5 shows the microstructure of the as-cast CoCrFeNi alloy (Figure 5a) and those annealed at 500 °C and 900 °C (Figure 5b,c, respectively). All images show a homogeneous structure without any inclusions, which corresponds well to the XRD analysis results. In the case of this alloy, annealing did not change the microstructure.

**Figure 6:**
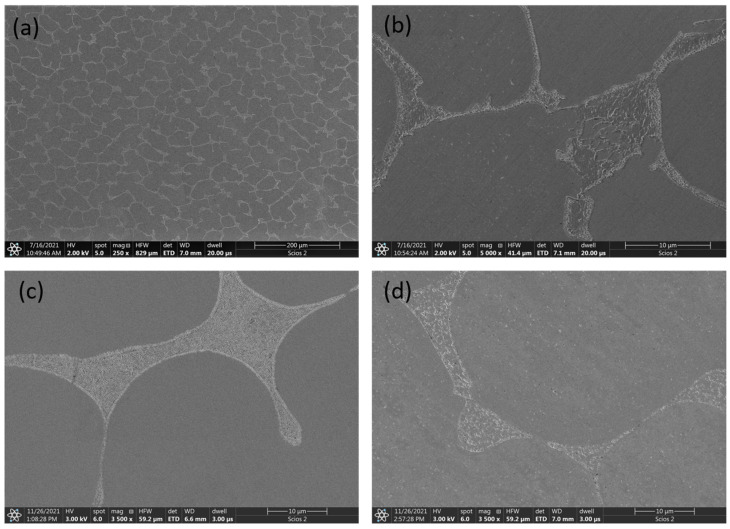
SEM micrographs of Al_0.5_CoCrFeNi alloy: (**a**,**b**) as-cast, (**c**) annealed at 500 °C, and (**d**) annealed at 900 °C.

The microstructure of the Al_0.5_CoCrFeNi alloy is biphasic. At lower magnification, there are clearly visible dendrites of the phase that is a solid solution of the constituent elements (Figure 6a), while a mixture of phases can be observed in the interdendritic spaces (Figure 6b). The dendritic areas are poorer in aluminum, while the interdendritic spaces have a significantly higher aluminum content. Annealing did not significantly affect the quantitative ratio of dendritic areas and interdendritic spaces (Figure 6c,d). Only in the case of sample 2.2 did the image of the interdendritic mixture change after annealing at 900 °C—there was a coagulation of the light phase precipitates, which is visible as spherical precipitates (Figure 6d).

**Figure 7:**
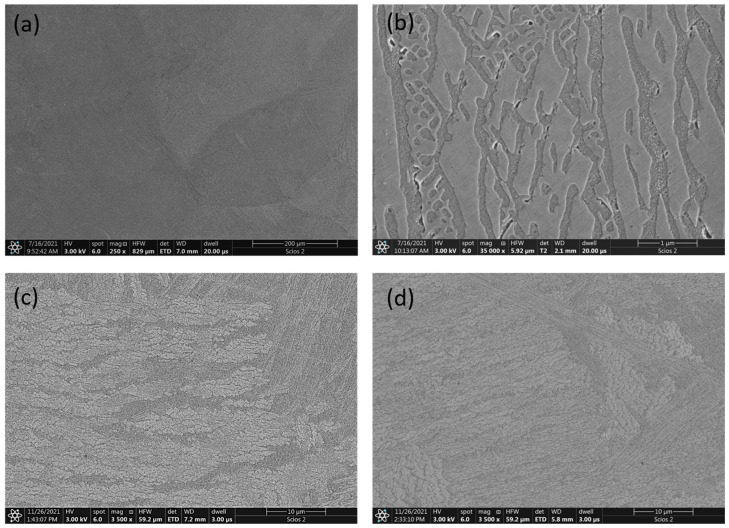
SEM micrographs of Al_0.7_CoCrFeNi alloy: (**a**,**b**) as-cast, (**c**) annealed at 500 °C, and (**d**) annealed at 900 °C.

The images in Figure 7 show the microstructures of the series 3 alloys before and after heat treatment. In this case, a two-phase structure is also visible. The arrangement of the areas of the individual phases is not dendritic, as in the case of series 2 alloys; it is more like a lamellar Widmanstatten structure (Figure 7a–d). It should be noted that the arrangement and shape of the two phases’ precipitates are similar for all samples in this series.

Table 3 shows the results of the chemical composition analysis of the tested as-cast alloys. The average values obtained from the larger measurement plots correspond approximately to the assumed values of the constituent elements. In the case of alloys with aluminum contents of x = 0.5 and x = 0.7, due to the two-phase structure, the chemical composition of the main phase (dendrite or side plate) and the spaces between the separations of this phase were also analyzed. It can be seen that there are convergences between these results. The dendrite (x = 0.5) and inter-sideplate (x = 0.7) phases are richer in aluminum compared to the averaged values for these materials. In contrast, the interdendrite and sideplate phases are poorer in aluminum. Note that the chemical composition of the separated phases is similar for both alloys (x = 0.5 and x = 0.7), only the quantitative contribution of these phases is different (Figure 6 and Figure 7).

### 3.3. Mechanical Properties

**Figure 8:**
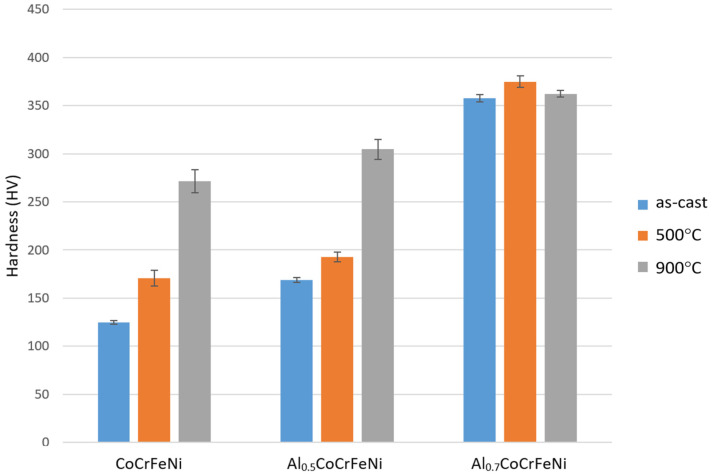
The average hardness of obtained alloys.

The effect of heat treatment on the selected mechanical properties of the obtained alloys was also analyzed. The results of the alloy hardness measurements are shown in Figure 8. With increasing aluminum content, the hardness of the alloy becomes greater. The received results are consistent with other studies [15,31]. The reason for such a large increase in hardness may be the phases occurring in the alloy. An increase in the aluminum concentration in the alloy leads to the change of the fcc phase into a mixture of the fcc and bcc, and then into the bcc.

In the case of CoCrFeNi and Al_0.5_CoCrFeNi alloys, the heat treatment had a bigger influence on the hardness than in the Al_0.7_CoCrFeNi alloy. Annealing at 900 °C increased the hardness of the CoCrFeNi alloy by 218% and the Al_0.5_CoCrFeNi alloy by 181% compared to the initial alloys. In the case of these two materials (series 1 and 2), annealing at 500 °C resulted in a significantly lower increase in hardness than annealing at 900 °C. In the series 3 alloy, Al_0.7_CoCrFeNi, a slightly different result was observed. The heat treatment had a smaller influence on properties. Annealing at 500 °C resulted in an increase in hardness of only 10%, while at 900 °C the increase is minimal. Similar results for the Al_0.75_CoCrFeNi alloy were obtained by the authors of [27].

**Figure 9:**
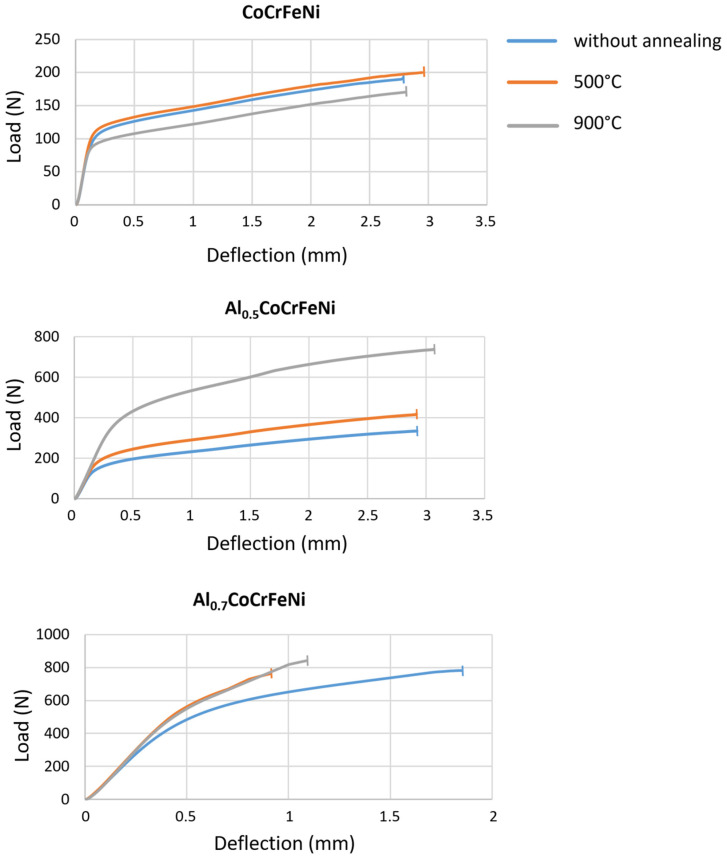
Averaged load–displacement curves.

Figure 9 shows the averaged load–displacement curves determined from the results received during the three-point bending test.

**Figure 10:**
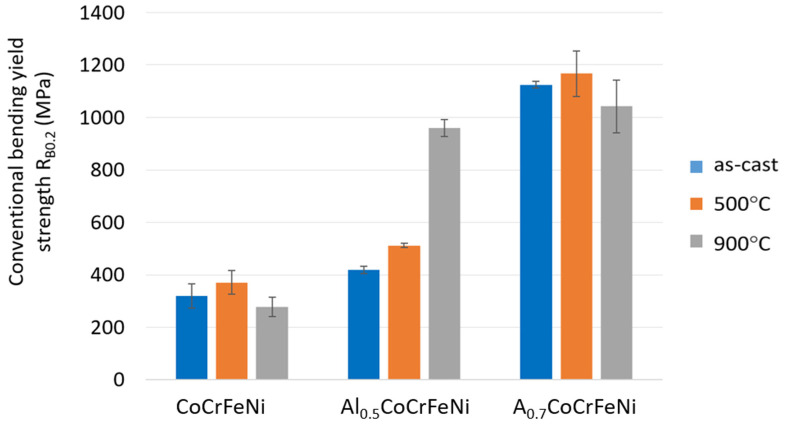
Determined conventional yield strength R_0.2_.

**Figure 11:**
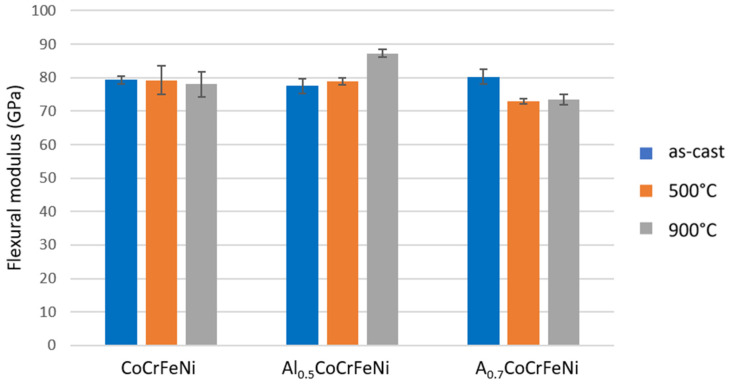
Determined flexural modulus.

Selected alloy properties determined from the three-point bending test are shown in Figure 10 and Figure 11.

Figure 10 shows that annealing at 500 °C increased the conventional bending yield strength of all samples. In this case, the flexural modulus after heat treatment at 500 °C did not change for the CoCrFeNi sample, but increased for Al_0.5_CoCrFeNi and decreased significantly for Al_0.7_CoCrFeNi (Figure 5). Heat treatment at 900 °C resulted in a large increase in the conventional bending yield strength of the Al_0.5_CoCrFeNi sample, while for the other samples the obtained values were lower than before annealing. The same is observed for the determined flexural modulus.

### 3.4. Correlation between Structure and Mechanical Properties

The results of the study clearly showed the significant influence of the aluminum content in the Al_x_CoCrFeNi alloy and the annealing process at different temperatures on the mechanical properties. Which strengthening mechanism plays the greatest role here, solution strengthening, related to the difference in atomic radii of aluminum compared to the other constituent elements, phase transformations of the fcc and bcc structure with different mechanical properties, or interactions at the microstructural level?

Solution strengthening should play an important role, as confirmed by the authors of [32]. It could be expected that an increase in aluminum content from x = 0 to x = 0.5 would result in greater strengthening than between alloys x = 0.5 and x = 0.7. Comparing the data shown in Figure 8 and Figure 10, it can be seen that the properties of the Al_0.5_CoCrFeNi alloy are slightly higher than those of CoCrFeNi. In contrast, at an aluminum content of x = 0.7, both hardness and yield strength increase by more than 100% compared to the alloy without aluminum.

Many authors believe that the change in the crystal structure is mainly responsible for the strengthening of Al_x_CoCrFeNi alloys. Usually, the mixing enthalpy (H_mix_) and the valence electron concentration (VEC) are employed to predict the stability of fcc and bcc solid solutions in these alloys. According to calculations [32,33] at low aluminum content there is a homogeneous fcc phase, over 10% of the aluminum atomic content a bcc phase appears, and its proportion increases with the aluminum concentration. A single-phase bcc structure occurs for x = 1. This is confirmed by the XRD observations of many researchers [34]. Certainly, the phase structure affects the mechanical properties of the alloys. According to the results of the authors of [34], the hardness of the Al_x_CoCrFeNi alloy improves from x = 0 to x = 0.9, while a further increase in aluminum content to x = 1.5 does not affect the hardness growth. In addition, heat treatment of materials also causes phase transformations, e.g., for x = 0.5 above 800 °C a bcc → fcc transformation occurs [35]. The authors of [24] observe that annealing at 600 °C causes an increase in the contribution of the bcc phase, resulting in improved hardness. However, heat treatment above 800 °C causes a decrease in hardness, which they justify by the separation of Al and Ni from the fcc matrix. Many authors point out the strong influence of the σ phase on the strengthening of the AlCoCrFeNi alloy [22,28]. This phase appears during annealing at 800 °C, and it disappears at temperatures above 1000 °C. However, it only occurs at higher aluminum contents (above x = 0.9) [25,34]. Based on the data obtained in the present work, it is difficult to justify the changes in strength properties on the basis of phase transformations. In the case of the CoCrFeNi alloy (x = 0), practically no changes were observed in the crystalline structure and microstructure due to annealing (single-phase fcc structure). There was also no change in the bending yield strength. However, this does not correlate with the change in hardness—after annealing at 900 °C, the hardness increased more than twice compared to the initial material. Thermal stresses were unlikely to affect the properties—the alloy was cooled slowly with the furnace. For an alloy with an aluminum content of x = 0.5, a good correlation of the hardness and yield strength results was obtained: after annealing at 500 °C, these parameters increased by about 14% and 22%, respectively, while a significant increase of 81% and 129% was obtained after annealing in 900 °C. In the case of this alloy, it could be justified by the presence of a hard bcc phase, which appears with increasing aluminum content, but quantitative XRD studies do not confirm the increased proportion of the bcc phase after heat treatment at 900 °C compared to the initial material.

It appears that the most possible connection can be made between the changes in mechanical properties and the microstructure of the investigated alloys obtained by induction melting. In the case of the series 1 alloy (without aluminum), we are considering a single-phase structure. Annealing had almost no effect on the change in microstructure and yield strength values. It is interesting that the hardness of this alloy increased with the annealing temperature, which does not correspond to the parameter *R*_*B*0.2_. In contrast, for the series 2 alloy (x = 0.5) the results of hardness and *R*_*B*0.2_ measurements are analogous—there was a significant increase in mechanical properties after annealing at 900 °C. This can be connected with changes in the microstructure of the alloy (Figure 6). The microstructure of the investigated Al_0.5_CoCrFeNi alloy consists of dendrites of the fcc phase which are deficient in aluminum, and a small proportion of interdendritic spaces with a mixture of phases with increased aluminum content. It is possible to observe slight changes in the microstructure in the interdendrite areas after annealing at 900 °C (Figure 6d). This may be caused by the migration of atoms at elevated temperatures and the appearance of precipitates at phase boundaries in interdendritic spaces. Explaining this aspect requires more detailed research. The authors of [35] suggest an effect of coherent nanoparticles on the strengthening of the AlCrFeNiV alloy. In contrast, the Al_0.7_CoCrFeNi alloy shows the highest mechanical properties regardless of the additional annealing, even though in this case the heat treatment had a significant effect on the changes in the crystal structure. In the microstructure of this alloy, the phases that are richer and poorer in aluminum are evenly distributed over the entire surface of the sample, which was hardly affected by annealing.

The mechanism of strengthening in Al_x_CoCrFeNi alloys with increasing Al content during annealing is complex and requires further detailed analyses with complex consideration of phase and microstructural transformations. Nevertheless, the effect is very significant, which raises the importance of such studies.

It should be mentioned that the results presented by other researchers investigating similar alloys are also often inconclusive, particularly regarding the mechanical properties and their changes under annealing. This fact is well illustrated by a summary prepared by the authors [22] for the equimolar alloy AlCoCrFeNi. In addition, significant differences are observed depending on the way the material is prepared (mechanical alloying, SPS [36], induction melting, which the authors used, laser deposition [32], or plasma sprayed in the case of layer deposition [24]).

## 4. Conclusions

The research presented in this article analyzes the effect of annealing at 500 °C and 900 °C of selected Al_x_CoCrFeNi alloys obtained by induction melting. The following conclusions can be drawn from the achieved results:

- No significant changes in the microstructure of the studied alloys were observed as a result of the heat treatment. The alloy without aluminum was characterized by a single-phase structure. However, in the microstructure of alloys with an aluminum content of x = 0.5 and x = 0.7, the presence of two phases differing in aluminum content can be observed.
- Changes in the crystalline structure of the alloys are related to changes in the aluminum content. The addition of aluminum enhances the appearance of the bcc phase. In the case of CoCrFeNi and Al_0.5_CoCrFeNi alloys, annealing at 500 °C and 900 °C did not significantly affect the changes in the crystalline structure. On the other hand, a significant effect of annealing on the crystalline structure of the Al_0.7_CoCrFeNi alloy was noted. By annealing at 900 °C, a permanent transformation to fcc occurred.
- These transformations are not reflected in changes in the mechanical properties of the tested materials. In the case of CoCrFeNi and Al_0.5_CoCrFeNi alloys, annealing at 900 °C resulted in a strengthening of the materials during the three-point bending test. In contrast, no significant changes in mechanical properties were observed for the Al_0.7_CoCrFeNi alloy as a result of annealing.
- As the aluminum content increases, the mechanical properties of the Al_x_CoCrFeNi alloys improve, particularly at an aluminum content of x = 0.7.

## Figures and Tables

**Table 1:** List of Al_x_CoCrFeNi alloy specimens used for testing.

No Series	Al Content	Alloy	Annealing
1.0	x = 0	CoCrFeNi	as-cast
1.1			500 °C
1.2			900 °C
2.0	x = 0.5	Al_0.5_CoCrFeNi	as-cast
2.1			500 °C
2.2			900 °C
3.0	x = 0.7	Al_0.7_CoCrFeNi	as-cast
3.1			500 °C
3.2			900 °C

**Table 2:** Phase analyses carried out by the use of the ICSD database with respect to referenced cubic space groups number, the unit cell parameters, and the percentage of phase volume contribution. Vol % obtained from XRD data.

Sample		Crystal Structure Data	Vol. %	Graphic Symbol	Crystallite Size (nm)
CoCrFeNi	as-cast	3.6524(3) Å/Fm-3m no. 225	19	ordered fcc/F_1_	3009.7
		3.5644(1) Å/Fm-3m no. 225	81	ordered fcc/F_2_	
	500 °C	3.5768(2) Å/Fm-3m no. 225	100	ordered fcc/F_2_	41.9
	900 °C	3.6022(2) Å/Fm-3m no. 225	18	ordered fcc/F_1_	86.3
		3.5712(1) Å/Pm-3m no. 221	82	disordered fcc/F_2d_	
Al_0.5_CoCrFeNi	as-cast	2.8609(3) Å/Im-3m no. 229	9	ordered bcc/I	29.8
		3.5915(1) Å/Fm-3m no. 225	91	ordered fcc/F_2_	37.3
	500 °C	2.8637(1) Å/Im-3m no. 229	3	ordered bcc/I	
		3.5796(1) Å/Pm-3m no. 221	97	disordered fcc/F_2d_	34.2
	900 °C	2.8642(1) Å/Im-3m no. 229	11	ordered bcc/I	41.2
		3.5707(2) Å/Pm-3m no. 221	89	disordered fcc/F_2d_	37.2
Al_0.7_CoCrFeNi	as-cast	2.8584(5) Å/Im-3m no. 229	6	ordered bcc/I	31.3
		3.5948(1) Å/Fm-3m no. 225	94	ordered fcc/F_2_	51.1
	500 °C	2.8612(2) Å/Im-3m no. 229	54	ordered bcc/I	33.4
		3.5926(3) Å/Fm-3m no. 225	46	ordered fcc/F_2_	58.5
	900 °C	3.6578(4) Å/Fm-3m no. 225	1	ordered fcc/F_1_	59.8
		3.5750(1) Å/Pm-3m no. 221	89	disordered fcc/F_2d_	
	disordered I/I_d_	5.7335(8) Å/Fm-3m no. 225	3	ordered L2_1_/L	
		3.6054(3) Å/Pm-3m no. 221	7	disordered fcc/F_3d_	

**Table 3:** Chemical composition of obtained alloys, EDX analysis.

Alloys	Area	Element (at. %)				
		Al	Co	Cr	Fe	Ni
CoCrFeNi	global	-	24.82	24.50	25.59	25.09
Al_0.5_CoCrFeNi	global	10.96	22.72	21.27	23.01	22.04
	dendrite	9.18	23.81	21.66	24.83	20.52
	interdendrite	16.71	19.03	22.96	19.12	22.19
Al_0.7_CoCrFeNi	global	13.82	21.86	21.40	22.13	20.79
	side plate	10.45	22.89	22.11	23.85	20.71
	inter-sideplate	16.25	19.75	23.28	21.17	19.55

## Data Availability

The data presented in this study are available on request from the corresponding author.
